# The real cost of sequencing: scaling computation to keep pace with data generation

**DOI:** 10.1186/s13059-016-0917-0

**Published:** 2016-03-23

**Authors:** Paul Muir, Shantao Li, Shaoke Lou, Daifeng Wang, Daniel J Spakowicz, Leonidas Salichos, Jing Zhang, George M. Weinstock, Farren Isaacs, Joel Rozowsky, Mark Gerstein

**Affiliations:** Department of Molecular, Cellular and Developmental Biology, Yale University, New Haven, CT 06520 USA; Systems Biology Institute, Yale University, West Haven, CT 06516 USA; Integrated Graduate Program in Physical and Engineering Biology, Yale University, New Haven, CT 06520 USA; Program in Computational Biology and Bioinformatics, Yale University, New Haven, CT 06520 USA; Department of Molecular Biophysics and Biochemistry, Yale University, New Haven, CT 06520 USA; The Jackson Laboratory for Genomic Medicine, Farmington, CT 06032 USA; Department of Computer Science, Yale University, New Haven, CT 06520 USA

## Abstract

As the cost of sequencing continues to decrease and the amount of sequence data generated grows, new paradigms for data storage and analysis are increasingly important. The relative scaling behavior of these evolving technologies will impact genomics research moving forward.

## History from the 50s to next generation sequencing

**Fig. 1:**
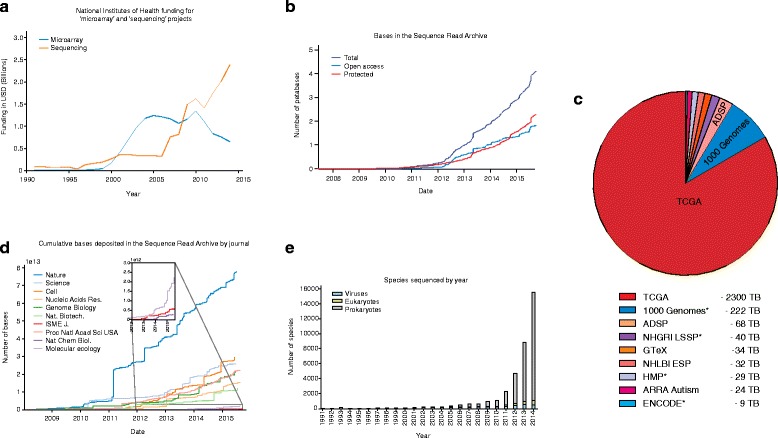
The dramatic increase in the rate and amount of sequencing. **a** Next generation sequencing (NGS) reads have become the dominant form of sequence data. This is illustrated in a graph of National Institutes of Health (NIH) funding related to the keywords “microarray” and “genome sequencing”, which shows increasing funding for NGS and decreases in the funding of earlier technologies such as microarrays. **b** The size and growth rate of the Sequence Read Archive (SRA) highlight the importance of efficiently storing sequence data so that they can be accessed by the broader scientific community. The SRA’s centrality in the storage of DNA sequences from next-generation platforms means that it also serves as a valuable indicator of the scientific uses of sequencing. Furthermore, the rise in protected sequence data highlights the challenges facing genomics as ever-greater amounts of personally identifiable sequence data are being generated. **c** It is interesting to look at the contribution of large sequence depositions compared to smaller submissions. This provides an indication of the size distribution of sequencing projects. At one end of this size spectrum are large datasets generated by the collaborative effort of many labs. These include projects that have taken advantage of sequencing trends to generate population-scale genomic data (1000 Genomes) or extensive characterization of cancer genomes by The Cancer Genome Atlas (TCGA). On top of generating a vast amount of sequencing data with the aim of better understanding human variation and disease, high-throughput sequencing has dramatically expanded the number of species whose genomes are documented. The number of newly sequenced genomes has exhibited an exponential increase in recent years. Entries with asterisks indicate projects that produce open access data. ADSP, Alzheimer’s Disease Sequencing Project; HMP, Human Microbiome Project. **d** A more detailed analysis of the SRA illustrates the pace at which different disciplines adopted sequencing. Plots depicting the cumulative number of bases deposited in the SRA and linked to papers appearing in different journals provide a proxy for sequencing adoption. More general journals such as *Nature* and *Science* show early adoption. Meanwhile, SRA data deposited by articles from more specific journals such as *Nature Chemical Biology* and *Molecular Ecology* remained low for a relatively long period before increasing. These trends highlight the spread of sequencing to new disciplines. **e** Sequence data have also been distributed over the tree of life. In terms of size, the vast majority of sequence data generated have been for eukaryotes. This is due in part to the larger genome size of eukaryotes and to efforts to sequence multiple individuals within a given species, especially humans. In terms of the number of species sequenced, prokaryotes are by far the best represented. Moving forward, the continuing decrease in the cost of sequencing will enable further exploration of genetic diversity both within and across species. Data were obtained from GenBank

In the 1950s, the contemporaneous development of biopolymer sequencing and the digital computer started a digital revolution in the biosciences. Then in the late 1970s, the advent of the personal computer (PC) and Sanger sequencing led to an appreciable amount of sequence data being generated, stored in databases, and conceptualized within a computational framework [1–4]. Communal sequence databases were developed in the 1980s [5, 6], but most investigators worked with data of a scale that allowed transfer to and processing on a local client. In the 1990s, the rise of the Internet facilitated increased data sharing, and analysis techniques began to shift to programs hosted on websites [7]. In the mid-2000s, the most recent big change occurred with the advent of cloud computing and next generation sequencing (NGS), which led to a dramatic increase in the scale of datasets (Fig 1) [4, 8]. This necessitated changes in the storage infrastructure; databases such as the European Nucleotide Archive [9] and the Sequence Read Archive (SRA) [10] were created to store and organize high-throughput sequencing data. The SRA has grown significantly since its creation in 2007, and it now contains almost four petabases (4 × 10^15^ bases), approximately half of which are open access [11]. These datasets present a challenge because they are too large for the old sharing and analysis paradigms, but recent innovations in computational technologies and approaches, especially the rise of cloud computing, provide promising avenues for handling the vast amounts of sequence data being generated.

## Organizing principles for biocomputing history

There are a number of key concepts to keep in mind when considering the coevolution of sequencing and computing. First is the idea that scientific research and computing have progressed through a series of discrete paradigms driven by the technology and conceptual frameworks available at the time, a notion popularized by Jim Gray from Microsoft [12]. Gray organized his views into four paradigms of scientific research. The first two paradigms are empirical observation and attempts to identify general theories. Gray’s third paradigm describes the original type of scientific computing, epitomized by large supercomputer-based calculations and modeling, for example, computing a rocket trajectory from a set of equations. This approach tends to favor differential equations and linear-algebraic types of computations.

The fourth paradigm is much more data intensive. Here the “capture, curation, and analysis” of large amounts of information fuels scientific research [12]. Researchers often try to find patterns in “big data” and a premium is placed on resource interoperability and statistical pattern finding. In order to realize fully the potential of this approach to science, significant investment must be made both in the computational infrastructure that supports data processing and sharing and in providing training resources that will allow researchers to better understand, handle, and compare large datasets.

The second key concept is the interplay between fixed and variable costs, especially with regard to their impact on scaling behavior. Much of the decrease in sequencing costs has been a result of a shift between these two cost structures. NGS introduced more efficient and complicated equipment, increasing the fixed cost; but a reduction of the variable costs of sequencing resulting from lower per-sample costs has accompanied this increase in fixed cost. This has encouraged the sequencing of an ever-greater number of samples in order to reduce the average cost and achieve economies of scale.

The opposite shift in cost structures is beginning to occur in the context of scientific computing. In the past, computing operated under a cost structure similar to that for sequencing. This often involved a large fixed cost associated with purchasing a machine followed by low variable costs for actual running of the machine (usually power, cooling, and systems administration time). Cloud computing and its associated concepts, such as the software, platform, and infrastructure as a service, removes the need for a large initial fixed-cost investment [13]. However, the variable costs associated with access to cloud computing can be significantly higher. This new regime, in which costs scale with the amount of computational processing time, places a premium on driving down the average cost by developing efficient algorithms for data processing.

The different cost structure of this new computing paradigm will significantly impact how funding agencies and researchers approach data analysis. Traditionally, large expenses for computing equipment in academic settings have been exempt from additional indirect fees levied by universities on smaller consumption purchases. Furthermore, the running costs for the hardware, such as electricity and cooling costs, are supported by the university at little to no cost for the individual investigator (usually from the overall pool of indirect costs). By contrast, universities do not consider cloud computing time to be an equipment purchase and levy the indirect cost fees on top of the ‘service’ purchase. In addition, cloud computing costs often incorporate the additional costs (electricity, rent, and so on) directly into the price. These funding schemes add to the expense of purchasing cloud-computing time as compared to large purchases of computing equipment.

The cost of sequencing is frequently measured as a dollar amount per base. Whether this price includes all steps in the sequencing process (sample preparation, downstream processing, and so on) or merely the sequencing run is often ambiguous. This single price also obscures the cost breakdown of sequencing projects. A more comprehensive approach in which the full economic cost (FEC) of sequencing is evaluated would enable both researchers and funding agencies to better understand and plan such projects. This approach breaks the cost of a sequencing project into its substituent parts and identifies the shared institutional resources used as well as the indirect costs associated with the project. Such accounting practices would more explicitly call attention to the shift in cost structures described above and would better enable the adaptation of funding mechanisms to meet the changing needs of sequencing-enabled research.

**Fig. 2:**
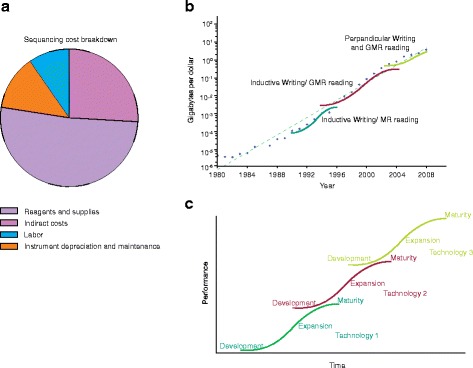
**a** The cost breakdown of next generation sequencing projects. The total cost of these projects is split into the cost of labor, reagents and supplies, instrument depreciation and maintenance, and indirect fees. **b** The exponential increase in the number of gigabytes per dollar in hard drive storage technology is due in part to the sequential introduction and improvement of three technologies. Data were obtained from http://www.mkomo.com/cost-per-gigabyte. **c** Exponential scaling in technological cost improvement is often the superposition of multiple S-curve trajectories of individual technologies. At the beginning of a technology’s life cycle, development costs keep cost reductions low. As the technology matures improvements in production are able to drive down per unit costs and establish an exponential regime. Eventually, the technology reaches maturity where technological limits are encountered and the cost improvements again slow down. GMR reading, Giant Magnetoresitive reading; MR reading, Magnetoresitive reading

Such detailed cost breakdowns are often difficult to obtain and can vary between institutions. Nevertheless, these cost breakdowns can help to reveal how different components of the sequencing pipeline scale with the size of the project. Figure 2a illustrates the cost breakdown of NGS projects into the costs of labor, reagents and supplies, instrument depreciation and maintenance, and indirect fees. These analyses have a common drawback in that they generally exclude bioinformatics costs or include only the cost of basic data processing (without alignment) and initial storage. As bioinformatics becomes increasingly important in the generation of biological insight from sequencing data, the long-term storage and analysis of sequencing data will represent a larger fraction of project cost. Efforts to better incorporate detailed and realistic accounting for downstream bioinformatics analysis is essential to the development of accurate models of the FEC of sequencing projects.

The third key concept to take into account with these developments is the idea of scaling behavior in sequencing technology and its impact on biological research. The most prominent analogous example of this is Moore’s law, which describes the scaling of integrated circuit development and its wide-ranging impact on the computer industry.

## Backdrop of the computer industry and Moore’s law

Improvements in semiconductor technology have dramatically stimulated the development of integrated circuits during the past half-century. This spurred the development of the PC and the internet era. Various scaling laws that model and predict the rapid developmental progress in high-tech areas driven by the progress in integrated circuit technology have been proposed. Moore’s law accurately predicted that the number of transistors in each square inch would double every two years [14]. In fact, the integrated circuit industry has used Moore’s law to plan its research and development cycles. Besides Moore’s law, various other predictive laws have been proposed for related high-tech trends. Rock’s law (also called Moore’s second law) predicted that the fixed cost of constructing an integrated circuit chip fabrication plant doubles about every four years [15]. Additionally, Kryder’s law describes the roughly yearly doubling in the area storage density of hard drives over the past few decades [16].

The roughly exponential scaling over a period of multiple decades described by these laws is not simply the scaling behavior of a single technology but rather the superposition of multiple S-curve trajectories. These curves represent the scaling of different technological innovations that contribute to the overall trend (Fig. 2). The S-curve behavior of an individual technology is the result of three main phases: development, expansion and maturity [17]. For example, the near yearly doubling of hard drive storage density over the past two and a half decades results from the superposition of the S-curves for five different basic storage technologies. This behavior is also seen for sequencing-based technologies.

The success of these predictive laws encouraged the development of forecasts for other emergent technologies, including sequencing. The cost of sequencing roughly followed a Moore’s law trajectory in the decade before 2008, but the introduction of NGS technologies caused costs to drop faster than would be expected by Moore’s law. Specifically, in the past five years, the cost of a personal genome has dropped to $4200 in 2015 from $340,000 in 2008 [18]. This departure from Moore’s law indicates that the transition between these technologies introduced a new cost-scaling regime.

## Computational component of sequencing—what’s happening in bioinformatics?

The decreasing cost of sequencing and the increasing number of sequence reads being generated are placing greater demand on the computational resources and knowledge necessary to handle sequence data. It is crucially important that as the amount of sequencing data continues to increase, these data are not simply stored but organized in a manner that is both scalable and easily and intuitively accessible to the larger research community. We see a number of key directions of change in bioinformatics computing paradigms that are adapting in response to the ever-increasing amounts of sequencing data. The first is the evolution of alignment algorithms in response to larger reference genomes and sequence read datasets. The second involves the need for compression to handle large file sizes, and especially the need for compression that takes advantage of domain knowledge that is specific to sequencing data to achieve better outcomes than those provided by more generic compression algorithms. The third change involves the need for distributed and parallel cloud computing to handle the large amounts of data and integrative analyses. The fourth change is driven by the fact that, in the future, a large amount of sequencing data will be private data, related to identifiable individuals; consequently, there is a need to put protocols in place to secure such data, particularly within a cloud-computing environment.

## Innovations underlying scaling in alignment algorithms

**Fig. 3:**
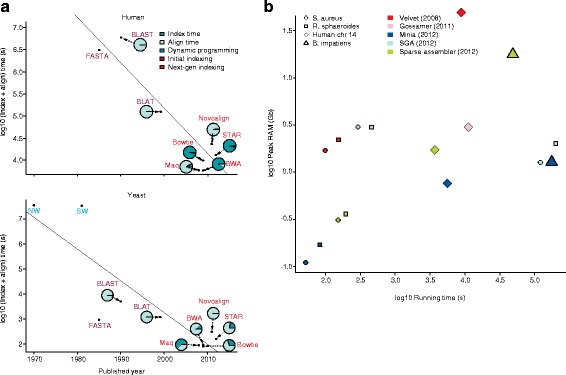
**a** Multiple advances in alignment algorithms have contributed to an exponential decrease in running time over the past 40 years. We synthesized one million single-ended reads of 75 bp for both human and yeast. The comparison only considers the data structure, algorithms, and speeds. There are many other factors, such as accuracy and sensitivity, which are not discussed here, but which are covered elsewhere [25]. Initial alignment algorithms based on dynamic programming were applicable to the alignment of individual protein sequences, but they were too slow for efficient alignment at a genome scale. Advances in indexing helped to reduce running time. Additional improvements in index and scoring structures enabled next generation aligners to further improve alignment time. A negative correlation is also observed between the initial construction of an index and the marginal mapping time per read. **b** Peak memory usage plotted against the running time for different genome assemblers on a log-log plot. Assembler performance was tested using multiple genomes, including *Staphylococcus aureus*, *Rhodobacter sphaeroides*, human chromosome 14, and *Bombus impatiens*. Data were obtained from Kleftogiannis et al. [33]

Alignment tools have co-evolved with sequencing technology to meet the demands placed on sequence data processing. The decrease in their running time approximately follows Moore’s Law (Fig. 3a). This improved performance is driven by a series of discrete algorithmic advances. In the early Sanger sequencing era, the Smith-Waterman [19] and Needleman-Wunsch [20] algorithms used dynamic programming to find a local or global optimal alignment. But the quadratic complexity of these approaches makes it impossible to map sequences to a large genome. Following this limitation, many algorithms with optimized data structures were developed, employing either hash-tables (for example, Fasta [21], BLAST (Basic Local Alignment Search Tool) [22], BLAT (BLAST-like Alignment Tool) [23], MAQ [24], and Novoalign [25]) or suffix arrays with the Burrows-Wheeler transform (for example, STAR (Spliced Transcripts Alignment to a Reference) [26], BWA (Burrows-Wheeler Aligner) [27] and Bowtie [28]).

In addition to these optimized data structures, algorithms adopted different search methods to increase efficiency. Unlike Smith-Waterman and Needleman-Wunsch, which compare and align two sequences directly, many tools (such as FASTA, BLAST, BLAT, MAQ, and STAR) adopt a two-step seed-and-extend strategy. Although this strategy cannot be guaranteed to find the optimal alignment, it significantly increases speeds by not comparing sequences base by base. BWA and Bowtie further optimize by only searching for exact matches to a seed [25]. The inexact match and extension approach can be converted into an exact match method by enumerating all combinations of mismatches and gaps.

In addition to changing search strategies, algorithms adjusted to larger datasets by first organizing the query, the database, or both. This involves an upfront computational investment but returns increased speed as datasets grow larger. For example, some algorithms (BLAST, FASTA, and MAQ) first build indexes for query sequences before scanning the database. On the database side, some algorithms (such as BLAST and MAQ) format the database into compact binary files, whereas others (such as BLAT, Novoalign, STAR, BWA, and Bowtie) build an offline index. STAR, BWA, and Bowtie in particular can significantly reduce the marginal mapping time (the time it takes to map a single read), but require a relatively large period of time to build a fixed index. In general, we find a negative correlation between the marginal mapping time and the time to construct the fixed index, making BWA, Bowtie, and STAR better suited to handle progressively larger NGS datasets (Fig. 3a). Much like the expansion phase observed in the S-curve trajectories that produce Moore’s law, many of these algorithms have been refined to improve performance. For example, BLAST has been heavily optimized for different datasets, producing HyperBLAST [29], CloudBLAST [30], DynamicBlast [31], and mBLAST [32], to name a few. In the case of mBLAST, researchers involved in the Human Microbiome Project commissioned the optimization of the algorithm so that the analyses could be performed on a reasonable time scale. Nevertheless, many of these alignment algorithms are not suitable for longer reads because of the scaling behavior of their seed search strategies. As long-read technologies continue to improve, there will be an ever greater need to develop new algorithms capable of delivering speed improvements similar to those obtained for short-read alignment [25].

Recently, new approaches have been developed that substitute assembly for mapping. These are not directly comparable to the mappers above, but they provide significant speed gains in certain contexts and may represent the next technological innovation in alignment. These approaches, including Salmon and Kallisto [29, 30], mostly focus on RNA-seq transcript identification and quantification, and they employ hashed k-mers and a De Bruijn graph for the task of RNA-Seq quantification. Moreover, instead of developing a base-pair resolution alignment, these approaches identify a ‘pseudoalignment’ that consists of the set of transcripts compatible with a given read.

In addition to read alignment, the other main computationally intensive algorithmic issue associated with the analysis of sequencing reads is the de novo assembly of a genome sequence. Many tools have been developed for assembly using short-read sequencing technology [31, 32]. The time and memory requirements are to some degree related to genome size but vary significantly between algorithms (Fig. 3b) [33]. The advent of long-read sequencing technologies such as Pacific Biosciences, Oxford Nanopore and Moleculo [34] promise high-quality sequence assemblies with potentially reduced computational costs. However, higher sequencing error rates for longer reads require novel assembly algorithms [35–38]. The main benefit is that it is possible to assemble contigs that are 10–100× larger than those assembled by traditional short-read technologies, even with lower-fold coverage (see [39] for a comparison in mammalian genomes).

## Compression

The explosion of sequencing data created a need for efficient methods of data storage and transmission. General algorithms such as Lempel-Ziv offer great compatibility, good speed and acceptable compression efficiency for sequencing data and are widely used [40], but customized algorithms are needed to further reduce the storage footprint and transmission time. For example, many researchers use the Sequence Alignment/Map (SAM)/Binary Alignment/Map (BAM) format to store reads. A widely accepted compression method, CRAM (compression algorithm), is able to shrink BAM files by ~30 % without any data loss (‘losslessly’) and by more if compression is allowed to lose some information (‘lossy’), typically in the quality scores [41]. CRAM only records the reference genome and applies Huffman coding to the result. The development of new and better compression algorithms is an active research field and we believe that high compatibility and the balance between usability and compression is key to moving forward.

## Cloud computing

Scalable storage, query, and analysis technologies are necessary to handle the increasing amounts of genomic data being generated and stored. Distributed file systems greatly increase the storage input/output (I/O) bandwidth, making distributed computing and data management possible. An example is the NoSQL database, which provides excellent horizontal scalability, data structure flexibility, and support for high-load interactive queries [42]. Moreover, the parallel programming paradigm has evolved from fine-grained MPI/MP to robust, highly scalable frameworks such as MapReduce [43] and Apache Spark [44]. This situation calls for customized paradigms that are specialized for bioinformatics study. We have already seen some exciting work in this field [45].

These distributed computing and scalable storage technologies naturally culminate in the framework of cloud computing, where data are stored remotely and analysis scripts are then uploaded to the cloud and the analysis is performed remotely. This greatly reduces the data transfer requirements because only the script and analysis results are transferred to and from data that reside permanently in the cloud.

## Privacy

Just as the internet gave rise to “open source” software, the initial sequencing of the human genome (particularly that from the “public consortium”) was associated with “open data”. Researchers were encouraged to build upon existing publicly available sequence knowledge and to contribute additional sequence data or annotations; but as more genomes of individuals are sequenced, concerns for the privacy of these subjects necessitates securing the data and providing access only to appropriate users [46].

As changing computing paradigms such as cloud computing become involved in managing the flood of sequencing data, privacy protection in the cloud environment becomes a major concern [47, 48]. Research in this field can broadly be split into two layers: first, sensitive data must be protected from leaking to a third party [49] and second, the cloud service provider should be made as oblivious as possible to the computation [50]. One possible culmination of these ideas could be the creation of a single, monolithic ‘biomedical cloud’ that would contain all the protected data from genomics research projects. This would completely change the biomedical analysis ecosystem, with researchers gaining access to this single entry point and storing all their programs and analyses there. Smaller implementations of this strategy can be seen in the development of Health Insurance Portability and Accountability Act (HIPAA)-compliant cloud resources, where datasets can be stored and shared on remote servers [48].

## The cost of sequencing and the changing biological research landscape

The decrease in the cost of sequencing that has accompanied the introduction of NGS machines and the corresponding increase in the size of sequence databases has changed both the biological research landscape and common research methods. The amount of sequence data generated by the research community has exploded over the past 10 years. Decreasing costs have enabled the formation of both large consortia with broad goals (such as measuring human genetic variation or profiling cancer genomes) and individual labs that target more specific questions. These developments have helped to democratize and spread sequencing technologies and research, increasing the diversity and specialization of experiments. Nearly 150 different experimental strategies have been described using Illumina sequencing alone. They apply this technology to nucleic acid secondary structure, interactions with proteins, spatial information within a nucleus, and more [51].

**Fig. 4:**
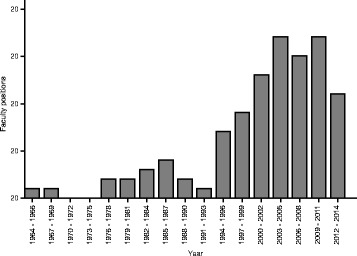
The number of faculty position hires at 51 US universities in 3-year bins. The recent increase in hiring coincides with the explosion in sequencing data. Data were obtained from http://jeffhuang.com/computer_science_professors.html

The changing cost structure of sequencing will significantly impact the social enterprise of genomics and bio-computing. Traditionally, research budgets have placed a high premium on data generation; but with sequencing prices falling rapidly and the size of sequence databases ever expanding, translating these data into biological insights is becoming increasingly important. Consequently, the analysis component of biological research is becoming a larger fraction of the real value of an experiment [8]. This of course shifts the focus of scientific work and the credit in collaborations. As a corollary, job prospects for scientists with training in computational biology remain strong, despite squeezed budgets [52]. Universities, in particular, have increased the number of hires in bioinformatics (Fig. 4).

Moreover, the falling price of sequencing and the growth of sequence databases has reduced the cost of obtaining useful sequence information for analysis. Sequence data that are downloadable from databases are ostensibly free, but costs arise in the need for computational storage and analysis resources as well as in the training necessary to handle and interpret the data. Initial automated processing pipelines for sequence data have lower fixed costs but higher variable costs compared to sequence generation. Variable costs associated with data transfer, storage, and initial pipeline processing using the cloud (such as to call variants) all scale with the size of the sequence dataset being analyzed. In sequence data generation, the high initial cost of a sequencing machine is offset by sequencing ever-greater amounts in order to distribute the cost of the initial capital investment over a larger number of sequenced bases, but this approach merely increases the amount of computational time required for initial pipeline processing. In the context of cloud computing, this translates into increasing costs because the user is charged for computational time used. This creates a mismatch: the combination of costs incurred in sequence data analysis are not subject to the same economy of scale seen in the generation of sequence data.

There are two possible cost structures for the downstream analysis, depending on how bioinformaticians are compensated. Bioinformaticians might be paid on a per project basis (in the extreme, an hourly wage) in which case their reimbursement resembles the low initial fixed cost and higher variable cost structure of cloud computing. On the other hand, if bioinformaticians are salaried, the cost structure of downstream analysis more closely resembles that of sequencing technologies, with the salaries representing an initial fixed cost. However, bioinformaticians differ from sequencing machines in that they cannot be consistently replaced by more expensive versions that are capable of processing more sequencing information. Consequently, driving down the cost of sequence analysis follows a similar path regardless of cost structure. In order to drive down costs, downstream analysis should be made as efficient as possible. This will enable bioinformaticians to analyze as much sequence data as possible under given time constraints. Generating ever-greater amounts of sequence information will become futile if those data hit a bottleneck during processing and analysis.

These factors necessitate that many of the big projects, in addition to generating large amounts of sequencing data, pay attention to making data analysis and processing efficient. This can often lead to a framework for large-scale collaboration in which much of the analysis and processing of the data is done in a unified fashion. This enables the entire dataset to be used as an enduring coherent resource that does not need reprocessing. If the sequence data generated by individual labs is not processed uniformly and sequence databases are not made easily accessible and searchable, then analysis of aggregated datasets will be challenging. It might seem superficially cheaper to pool the results of many smaller experiments but the reprocessing costs for all of these datasets may be considerably larger than redoing the sequencing experiment itself. In addition to posing technical issues for data storage, the increasing volume of sequences being generated presents a challenge in integrating newly generated information with the existing knowledge base. Hence, although people thought that the advent of NGS would democratize sequencing and spur a movement away from the large centers and consortia, in fact the opposite has been the case. The need for uniformity and standardization in very large datasets has, in fact, encouraged very large consortia such as 1000 Genomes [53] and The Cancer Genome Atlas (TCGA) [54].

In the future, one might like to see a way of encouraging uniformity and standardization without having an explicit consortium structure, letting many people aggregate small sequencing experiments and analyses together. Perhaps this could be done by open community standards just as the internet was built through pooling of many individual open-source actors using community-based standards [55]. It is imperative that such a standardization initiative accompanies the development and implementation of new technologies such as more efficient data processing and compression algorithms as well as secure cloud computing. A scalable biocomputing infrastructure is vital to a biological research ecosystem capable of integrating vast amounts of heterogeneous sequencing data.

## Abbreviations

BAM: Binary Sequence Alignment/Map
BLAST: Basic Local Alignment Search Tool
BLAT: BLAST-like Alignment Tool
BWA: Burrows-Wheeler Aligner
CRAM: Compression algorithm
FEC: Full economic cost
NGS: Next-generation sequencing
PC: Personal computer
SRA: Sequence Read Archive
STAR: Spliced Transcripts Alignment to a Reference.

## References

[1] Staden R (1982). Automation of the computer handling of gel reading data produced by the shotgun method of DNA sequencing. Nucleic Acids Res.

[2] Sanger F, Nicklen S, Coulson AR (1977). DNA sequencing with chain-terminating inhibitors. Proc Natl Acad Sci U S A.

[3] Larson R, Messing J (1983). Apple II computer software for DNA and protein sequence data. DNA.

[4] Stevens H (2013). Life out of sequence : a data-driven history of bioinformatics.

[5] George DG, Barker WC, Hunt LT (1986). The protein identification resource (PIR). Nucleic Acids Res.

[6] Kanehisa MI (1982). Los Alamos sequence analysis package for nucleic acids and proteins. Nucleic Acids Res.

[7] Gouet P, Courcelle E, Stuart DI, Metoz F (1999). ESPript: analysis of multiple sequence alignments in PostScript. Bioinformatics.

[8] Sboner A, Mu XJ, Greenbaum D, Auerbach RK, Gerstein MB (2011). The real cost of sequencing: higher than you think!. Genome Biol.

[9] Leinonen R, Akhtar R, Birney E, Bower L, Cerdeno-Tarraga A, Cheng Y (2011). The European Nucleotide Archive. Nucleic Acids Res.

[10] Leinonen R, Sugawara H, Shumway M (2011). International Nucleotide Sequence Database Collaboration. The sequence read archive. Nucleic Acids Res.

[11] Sequence Read Archive.NCBI/NLM/NIH. 2015. http://www.ncbi.nlm.nih.gov/Traces/sra/. Accessed 22 Feb 2016.

[12] Hey AJG, Tansley S, Tolle KM (2009). The fourth paradigm: data-intensive scientific discovery.

[13] Armbrust M, Fox A, Griffith R, Joseph AD, Katz R, Konwinski A (2010). A view of cloud computing. Commun ACM.

[14] Brock DC, Moore GE (2006). Understanding Moore’s law: four decades of innovation.

[15] Ross PE (2015). 5 Commandments.

[16] Walter C (2005). Kryder’s law. Sci Am.

[17] Sood A, James GM, Tellis GJ, Zhu J (2012). Predicting the path of technological innovation: SAW vs. Moore, Bass, Gompertz, and Kryder. Market Sci.

[18] National Human Genome Research Institute. DNA Sequencing Costs: Data from the NHGRI Genome Sequencing Program (GSP). http://www.genome.gov/sequencingcosts. Accessed 22 Feb 2016.

[19] Smith TF, Waterman MS (1981). Identification of common molecular subsequences. J Mol Biol.

[20] Needleman SB, Wunsch CD (1970). A general method applicable to the search for similarities in the amino acid sequence of two proteins. J Mol Biol.

[21] Lipman DJ, Pearson WR (1985). Rapid and sensitive protein similarity searches. Science.

[22] Altschul SF, Gish W, Miller W, Myers EW, Lipman DJ (1990). Basic local alignment search tool. J Mol Biol.

[23] Kent WJ (2002). BLAT— the BLAST-like alignment tool. Genome Res.

[24] Li H, Ruan J, Durbin R (2008). Mapping short DNA sequencing reads and calling variants using mapping quality scores. Genome Res.

[25] Li H, Homer N (2010). A survey of sequence alignment algorithms for next-generation sequencing. Brief Bioinform.

[26] Dobin A, Davis CA, Schlesinger F, Drenkow J, Zaleski C, Jha S (2013). STAR: ultrafast universal RNA-seq aligner. Bioinformatics.

[27] Li H, Durbin R (2009). Fast and accurate short read alignment with Burrows-Wheeler transform. Bioinformatics.

[28] Langmead B, Trapnell C, Pop M, Salzberg SL (2009). Ultrafast and memory-efficient alignment of short DNA sequences to the human genome. Genome Biol.

[29] Bray N, Pimentel H, Melsted P, Pachter L. Near-optimal RNA-Seq quantification. arXiv:150502710. 2015.

[30] Patro R, Duggal G, Kingsford C. Salmon: accurate, versatile and ultrafast quantification from RNA-seq data using lightweight-alignment. bioRxiv. 2015. http://dx.doi.org/10.1101/021592.

[31] Zhang W, Chen J, Yang Y, Tang Y, Shang J, Shen B (2011). A practical comparison of de novo genome assembly software tools for next-generation sequencing technologies. PLoS One.

[32] Bradnam KR, Fass JN, Alexandrov A, Baranay P, Bechner M, Birol I (2013). Assemblathon 2: evaluating de novo methods of genome assembly in three vertebrate species. Gigascience.

[33] Kleftogiannis D, Kalnis P, Bajic VB (2013). Comparing memory-efficient genome assemblers on stand-alone and cloud infrastructures. PLoS One.

[34] Kuleshov V, Xie D, Chen R, Pushkarev D, Ma Z, Blauwkamp T (2014). Whole-genome haplotyping using long reads and statistical methods. Nat Biotechnol.

[35] English AC, Richards S, Han Y, Wang M, Vee V, Qu J (2012). Mind the gap: upgrading genomes with Pacific Biosciences RS long-read sequencing technology. PLoS One.

[36] Koren S, Schatz MC, Walenz BP, Martin J, Howard JT, Ganapathy G (2012). Hybrid error correction and de novo assembly of single-molecule sequencing reads. Nat Biotechnol.

[37] Chin CS, Alexander DH, Marks P, Klammer AA, Drake J, Heiner C (2013). Nonhybrid, finished microbial genome assemblies from long-read SMRT sequencing data. Nat Methods.

[38] Lee H, Gurtowski J, Yoo S, Marcus S, McCombie WR, Schatz M. Error correction and assembly complexity of single molecule sequencing reads. bioRxiv. 2014. doi: http://dx.doi.org/10.1101/006395.

[39] Chaisson MJ, Wilson RK, Eichler EE (2015). Genetic variation and the de novo assembly of human genomes. Nat Rev Genet.

[40] Zhu Z, Zhang Y, Ji Z, He S, Yang X (2015). High-throughput DNA sequence data compression. Brief Bioinform.

[41] Hsi-Yang Fritz M, Leinonen R, Cochrane G, Birney E (2011). Efficient storage of high throughput DNA sequencing data using reference-based compression. Genome Res.

[42] Cattell R (2011). Scalable SQL, and NoSQL data stores. SIGMOD Rec.

[43] Dean J, Ghemawat S (2008). MapReduce: simplified data processing on large clusters. Commun ACM.

[44] Zaharia M, Chowdhury M, Franklin MJ, Shenker S, Stoica I (2010). Spark: cluster computing with working sets. Proceedings of the 2nd USENIX Conference on Hot Topics in Cloud Computing.

[45] Massie M, Nothaft F, Hartl C, Kozanitis C, Schumacher A, Joseph AD (2013). ADAM: genomics formats and processing patterns for cloud scale computing. Report No.: UCB/EECS-2013-207.

[46] Greenbaum D, Sboner A, Mu XJ, Gerstein M (2011). Genomics and privacy: implications of the new reality of closed data for the field. PLoS Comput Biol.

[47] Greenbaum D, Du J, Gerstein M (2008). Genomic anonymity: have we already lost it?. Am J Bioeth.

[48] Stein LD, Knoppers BM, Campbell P, Getz G, Korbel JO (2015). Data analysis: create a cloud commons. Nature.

[49] Popa RA, Redfield CMS, Zeldovich N, Balakrishnan H. CryptDB: protecting confidentiality with encrypted query processing. In: Proceedings of the twenty-third ACM symposium on operating systems principles. ACM; 2011. p. 85–100.

[50] Maas M, Love E, Stefanov E, Tiwari M, Shi E, Asanovic K, et al. PHANTOM: practical oblivious computation in a secure processor. Proceedings of the 2013 ACM SIGSAC conference on computer & communications security. ACM; 2013. p. 311–24.

[51] Illumina. A wide variety of library prep methods derived from the scientific literature. 2015. http://www.illumina.com/techniques/sequencing/ngs-library-prep/library-prep-methods.html. Accessed 22 Feb 2016.

[52] Levine AG (2014). An explosion of bioinformatics careers. Science.

[53] Auton A, Brooks LD, Durbin RM, Garrison EP, Kang HM, 1000 Genomes Project Consortium (2015). A global reference for human genetic variation. Nature.

[54] Weinstein JN, Collisson EA, Mills GB, Shaw KR, Ozenberger BA, Cancer Genome Atlas Research Network (2013). The Cancer Genome Atlas Pan-Cancer analysis project. Nat Genet.

[55] Isaacson W (2014). The innovators : how a group of hackers, geniuses, and geeks created the digital revolution.

