# Erratum to: The real cost of sequencing: scaling computation to keep pace with data generation

**DOI:** 10.1186/s13059-016-0961-9

**Published:** 2016-04-28

**Authors:** Paul Muir, Shantao Li, Shaoke Lou, Daifeng Wang, Daniel J. Spakowicz, Leonidas Salichos, Jing Zhang, George M. Weinstock, Farren Isaacs, Joel Rozowsky, Mark Gerstein

**Affiliations:** Department of Molecular, Cellular and Developmental Biology, Yale University, New Haven, CT 06520 USA; Systems Biology Institute, Yale University, West Haven, CT 06516 USA; Integrated Graduate Program in Physical and Engineering Biology, Yale University, New Haven, CT 06520 USA; Program in Computational Biology and Bioinformatics, Yale University, New Haven, CT 06520 USA; Department of Molecular Biophysics and Biochemistry, Yale University, New Haven, CT 06520 USA; The Jackson Laboratory for Genomic Medicine, Farmington, CT 06032 USA; Department of Computer Science, Yale University, New Haven, CT 06520 USA

After the publication of this work [1], the authors noticed an error in Fig. 4. The error occurred on the y-axis, instead of running from 0 to 20 it just has 20 at each point. The publisher apologises for the error and any inconvenience caused. The corrected figure is given below:

**Fig. 4:**
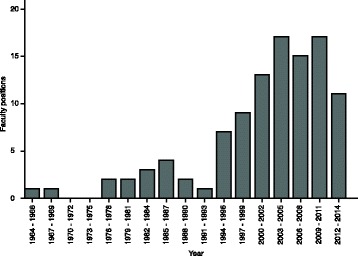
The number of faculty position hires at 51 US universities in 3-year bins. The recent increase in hiring coincides with the explosion in sequencing data. Data were obtained from http://jeffhuang.com/computer_science_professors.html
